# Learning, economies of scale, and knowledge gap effects on power generation technology cost improvements

**DOI:** 10.1016/j.isci.2024.111644

**Published:** 2024-12-19

**Authors:** Yoga W. Pratama, Matthew J. Gidden, Jenna Greene, Andrew Zaiser, Gregory Nemet, Keywan Riahi

**Affiliations:** 1 International Institute for Applied Systems Analysis, Laxenburg, Lower Austria, Austria; 2 Nelson Institute Center for Sustainability and the Global Environment, University of Wisconsin – Madison, Madison, WI, USA; 3 La Follette School of Public Affairs, University of Wisconsin – Madison, Madison, WI, USA

**Keywords:** Applied sciences, Engineering, Natural sciences

## Abstract

Cost reductions are essential for accelerating clean technology deployment. Because multiple factors influence costs, traditional one-factor learning models, solely relying on cumulative installed capacity as an explanatory variable, may oversimplify cost dynamics. In this study, we disentangle learning and economies of scale effects at unit and project levels and introduce a knowledge gap concept to quantify rapid technological change’s impact on costs. Our results show that a substantial proportion of cost declines in several technologies is attributable to economies of scale rather than learning processes. Thus, relying on one-factor learning may underestimate cost declines during upscaling periods for technologies with strong economies of scale effects and overestimate reductions for those approaching maximum size. Notably, the knowledge gap concept can endogenously capture how rapidly technology sizes can evolve through learning. These insights can improve decision-making and highlight the benefits of separating learning and economies of scale effects to estimate technology costs.

## Highlights

• Separating economies of scale from learning effects better explains cost dynamics
• Harnessing economies of scale effects can accelerate technology cost reductions
• The knowledge gap concept can capture how quickly technology sizes can change
• Relying on one-factor learning may underestimate cost declines for new technologies

## Graphical abstract

**Figure:**
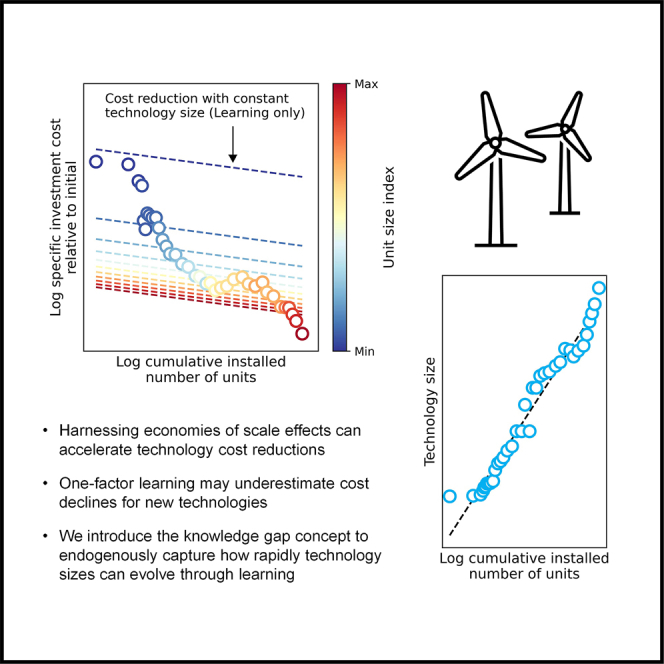

## Introduction

In order to limit global temperature rise to well below 2°C, clean energy technologies need to be rapidly deployed and displace existing CO_2_-emitting energy infrastructure. However, these clean technologies require cost reductions in their manufacturing, construction, and operation to make them cost-competitive with fossil fuel alternatives.^1^^,^^2^^,^^3^^,^^4^^,^^5^^,^^6^^,^^7^ Accordingly, identifying strategies, likely build-out needs, and required policy support to accelerate cost reductions is essential.

Technological innovation theory and empirical evidence point to a number of mechanisms by which cost reduction can be achieved. These factors include learning-by-doing (accumulating experience through increasing the number of deployments), learning-by-searching (via research, demonstration, and development/RD&D), learning-by-using, economies of scale (scaling up manufacturing, unit, and project size), manufacturing location (moving production to lower cost locations), and knowledge spillover.^8^^,^^9^^,^^10^^,^^11^^,^^12^^,^^13^^,^^14^^,^^15^^,^^16^ The interplay between learning by doing and economies of scale is particularly relevant. While economies of scale reduce costs at the unit and project levels, this might impede the learning progress because the need for fewer units and projects means the system will gain less experience in building them due to fewer iterations. In some cases, increasing sizes may actually increase costs due to the knowledge gap effect when the scale of technology changes too quickly, which might be caused by technical, logistical, and/or socio-economic challenges.^17^ As a result, quantifying the trade-offs between these factors is crucial to understanding pathways for cost reductions.

The concept of technology cost learning (also known as Wright’s Law) is well established, supported by literature offering parameter data^18^^,^^19^^,^^20^ and model implementation analysis.^21^^,^^22^^,^^23^^,^^24^^,^^25^^,^^26^^,^^27^^,^^28^^,^^29^ Most studies have focused on one-factor learning,^5^^,^^7^^,^^8^^,^^9^^,^^11^^,^^21^^,^^23^^,^^24^^,^^29^^,^^30^^,^^31^^,^^32^^,^^33^^,^^34^^,^^35^^,^^36^ using cumulative capacity as an explanatory variable to aggregate all factors involved in cost changes. This is partly due to scarce literature providing parameters for a more granular analysis in energy-economy models.^14^^,^^30^^,^^37^^,^^38^ Numerous studies indicate that cumulative capacity is a weak explanatory variable for essential factors influencing cost reductions.^7^^,^^8^^,^^11^ Additionally, despite its simplicity, such an approach offers limited insights into the contributions of each factor to cost change and how the progress can be accelerated.

Economies of scale can significantly reduce technology costs.^7^^,^^8^^,^^12^^,^^39^^,^^40^ Mixing this factor in the one factor learning exaggerates learning effects.^32^ Separating this factor from aggregated learning is essential for understanding feasible and affordable strategies for stringent climate mitigation goals.^30^^,^^32^ Accordingly, it is encouraged to implement this concept to estimate technology costs in decision-making processes and energy-economy models. However, literature on the parameters required to separate economies of scale from learning effects is limited.^30^ Some studies, such as in,^12^^,^^15^^,^^41^^,^^42^^,^^43^ provide these parameters for only a limited number of technologies and emphasize the need for extended works across a broader range of technologies.^30^

To leverage economies of scale, particularly for technologies with a strong effect, technology sizes at unit and project levels tend to increase over time.^39^ However, as previously mentioned, increasing sizes beyond certain limits can lead to dis-economies of scale, where economic disadvantages arise from scaling up the technology. Coulomb and Neuhoff^39^ indicated that learning processes are necessary to further increase technology sizes. Therefore, understanding the relationship between learning processes and size upscaling rates is essential. Literature has attempted to model this relationship by deriving logistic functions to detail the evolution of technology sizes over time^44^^,^^45^ or used exogenous assumptions to limit technology size scale-up.^43^ This analysis is useful for examining the historical evolution of technology scale-up and understanding various stages of technology development. Nevertheless, similar to cost reduction analysis,^22^^,^^32^^,^^33^ implementing this exogenous timeseries estimate in energy-economy models can lead to a model artifact, where technology deployment is deferred to periods with substantial immediate technology size increase, neglecting the need for learning and initial investment to achieve this.

This work aims to bridge these gaps by quantifying trade-offs between factors that affect technology cost reductions in a least-cost energy system modeling framework. Specifically, we emphasize learning-by-doing and economies of scale effects at both the unit and project levels. This study contributes to the existing literature by providing data and methods to estimate the parameters for an extended range of technologies, particularly focusing on power generation technologies, given the substantial role the sector is expected to play in climate mitigation scenarios. To quantify the dynamics between learning processes and the rates of unit and project scale-up, we propose a concept that correlates how quickly the experience stock allows the scaling of unit and project sizes. This concept, called the knowledge gap effect, identifies the point where gains from economies of scale are negated. To our knowledge, this study is among the first, if any, to quantify this relationship. In this work, we show how the implementation of this concept, with minor additional data and effort, facilitates a better understanding of technology cost dynamics for estimating future technology costs, which is a key to improving technology cost representation in climate mitigation analysis using energy-economy models.

### Technology cost change in IAMs

Energy-economy models, including those within integrated assessment models (IAMs), are frequently used to quantify the impact of cost improvements on transition pathways of energy systems.^21^^,^^22^^,^^23^^,^^24^ They can also be used to identify strategies to accelerate cost reductions, depending on how these reductions are represented in the model. Most importantly, insights from IAMs are widely used to provide guidance for global climate mitigation efforts. However, the representation of technology cost dynamics in IAMs remains limited. For instance, several IAMs treat technology cost reductions as exogenous variables.^27^^,^^28^^,^^46^ Other models incorporate these endogenously, relying on the one-factor learning approach where cost reductions depend solely on cumulative installed capacity.^25^^,^^26^^,^^27^^,^^28^ Only a few models adopt a more advanced approach, employing two-factor learning that accounts for both cumulative installed capacity and cumulative R&D expenditure.^16^^,^^31^^,^^47^^,^^48^^,^^49^ Therefore, enhancing the representation of technology cost dynamics in these models is essential.

Table 1 summarizes how technology cost improvements are implemented in IAMs. As can be observed in the table, many models with recursive dynamic simulation or non-linear programming optimization modeling approaches consider technology cost improvements endogenously owing to the ability of such formulation to directly implement the learning formulation in the model. IMAGE and REMIND, for instance, are models with one-factor learning where technology cost is a function of cumulative installed capacity. IMAGE is a recursive dynamic model that represents cost improvements of several selected technologies exclusively using one-factor learning, while the costs of other technologies are assumed exogenously.^50^^,^^51^ To compare, in addition to the one-factor learning, REMIND also takes into account different levels of spillover effect.^14^^,^^56^ These include global learning with perfect spillover between all regions and local learning with no spillover. Additionally, it also includes formulations to incorporate multi-level learning approaches to capture the effects of global and local cost components learning and partial convergence of the global components. Other models, such as POLES and WITCH, implement two-factor learning,^53^^,^^54^^,^^55^^,^^57^ in which the impact of research and development (R&D) expenditures on technology costs is incorporated. In these models, perfect global spillover is assumed.

**Table 1:** Technology cost improvement implementation in IAMs

Framework	Modeling approach^a^	Learning approach	Technology innovation implementation	Reference
IMAGE	Recursive dynamics	One-factor	Endogenous	Detlef & van Vuuren^50^; Roelfsema et al.^51^; Wilson et al.^52^
POLES	Recursive dynamics	Two-factor	Endogenous	Kouvaritakis et al.^53^; Kouvaritakis et al.^54^; Criqui et al.^55^; Wilson et al.^52^
REMIND	NLP optimization	One-factor	Endogenous	Zhang et al.^14^; Bauer et al.^56^; Wilson et al.^52^
WITCH	NLP optimization	Two-factor	Endogenous	Bosetti et al.^57^; Wilson et al.^52^
AIM	LP optimization	Exogenous	Exogenous	Hibino et al.^58^; Matsuoka et al.^59^; Fujimori et al.^60^; Wilson et al.^52^
COFFEE	LP optimization	Exogenous	Exogenous	Tagomori^61^; Callegari et al.^62^; Rochedo et al.^63^; Rochedo^64^; Cunha et al.^65^; Müller-Casseres et al.^66^
GCAM	Recursive dynamics	Exogenous	Exogenous	Binsted et al.^67^; Binsted et al.^68^; Snyder et al.^69^; Wilson et al.^52^;^70^
TIAM	LP optimization	One-factor	Endogenous, reformulation as MIP Exogenous, iterative approach	Loulou & Labriet^27^; Loulou^28^
MESSAGE	LP optimization	Exogenous One-factor	Exogenous Endogenous, reformulation as MIP	Messner^25^; Healey & Grubler^45^; Huppmann et al.^46^; Grubler et al.^71^; Wilson et al.^52^

AIM, Asia pacific Integrated Model, COFFEE, COmputable Framework For Energy and the Environment; GCAM, Global Change Assessment Model; IMAGE, Integrated Model to Assess the Global Environment; POLES, Prospective Outlook on Long-term Energy Systems; REMIND, REgional Model of Investment and Development; WITCH, World Induced Technical Change Hybrid; MESSAGE, Model for Energy Supply Systems And their General Environmental impacts; TIAM, TIMES Integrated Assessment Model; LP, Linear Programming; NLP, Non-linear Programming.

a Refers to the modeling approach in the module where technologies are represented.

Conversely, models with linear programming (LP) optimization approach tend to set technology cost changes as exogenous variables. AIM and COFFEE, for instance, set technology advancements, such as efficiencies and costs, as exogenous variables.^58^^,^^59^^,^^60^^,^^61^^,^^62^^,^^63^^,^^64^^,^^65^^,^^66^^,^^67^^,^^68^^,^^69^ Endogenous representation of technology cost improvements leads to the presence of bi-linear terms between investment cost and new capacity variables, which cannot directly be implemented in linear programming-based models. One approach to consider the cost improvements endogenously in LP models is by reformulating the model into mixed-integer linear programming (MILP/MIP), which was implemented in a number of analysis in TIAM and MESSAGE.^25^^,^^26^^,^^27^^,^^28^ In MESSAGE, earlier attempt to incorporate technology cost learning adopted the one-factor learning.^25^ Later, an approach that separates learning and economies of scale effects was introduced to quantify trade-offs between the two factors.^71^

Similar to MESSAGE, technology cost improvements in TIAM are taken into account as one-factor learning by reformulating the model into an MILP problem.^27^^,^^28^ More recent applications of the model incorporate the concept via an iterative-based approach. Here, the technology cost learning module is soft-linked to TIAM to reevaluate technology cost assumptions and its deployment output until convergence criteria are achieved.^34^

### Learning and economies of scale concepts

In 1936, aerospace engineer Theodore P. Wright observed a concept now known as the Wright’s Law.^72^ It explains how productivity increases with experience which then emerge into a concept that explains technology costs reduction as cumulative output increases due to learning-by-doing processes.^13^ Using this concept, the investment cost of technology can be estimated using Equations 1 and 2.

(Equation 1) $$IC_{t}=IC_{Ref}\;{\left(\frac{{KQ}_{t}}{{KQ}_{Ref}}\right)}^{-\alpha }$$

(Equation 2) $${KQ}_{t}={KQ}_{t-1}+Q_{t}$$

As shown in the equation, the specific investment cost IC in period t is a function of the specific investment cost reference ${IC}_{Ref}$ and the accumulated experience represented by the ratio of cumulative installed capacity KQ in period t and the reference period. Here, a constant α is the learning parameter which explains technology progress- (PR) and learning rates (LR), as described in Equations 3 and 4. In this context, a progress rate of 85% or a learning rate of 15% would mean that the cost of new capacity decreases by 15% with each doubling of cumulative installed capacity.

(Equation 3) $$\mathrm{PR}=2^{-\alpha }$$

(Equation 4) $$LR=1-2^{-\alpha }$$

Despite this commonly implemented learning approach, i.e., the use of cumulative installed capacity as the explanatory variable, literature suggest that unit size correlate negatively with learning,^16^^,^^30^^,^^44^^,^^73^ hence, scaling down unit size of the technology improves technology learning rates.^30^ The reason behind this is that smaller technologies allow more repetitive and replicative experience that drives technology improvements.^44^^,^^73^ Given that the number of repetitions and experimentations can better represent knowledge accumulation than does capacity, our approach quantifies the learning effect using the cumulative installed number of units KN instead of the cumulative capacity deployment. As can be seen in Equation 5, cumulative installed capacity in Equation 1 is replaced by cumulative installed number of units (KN), with KN in period t is the sum of KN in previous period and newly installed units N at the present period (Equation 6). Here, Equation 7 shows that capacity addition in Equation 2 is the product of number of units and the size.

(Equation 5) $$IC_{t}=IC_{Ref}\;{\left(\frac{{KN}_{t}}{{KN}_{Ref}}\right)}^{-\alpha }$$

(Equation 6) $${KN}_{t}={KN}_{t-1}+N_{t}$$

(Equation 7) $$Q_{t}=N_{t}\;S_{t}$$

In contrast to the learning effect, economies of scale refer to cost advantages from increasing the unit size, project size or production capacity. These advantages might have a physical basis, among other factors. For instance, the economies of scale in engineering can relate to the square–cube law, i.e., the surface area of a vessel is the square of its dimensions, while the volume is the cube. To illustrate, the cost of a spherical vessel can be estimated based on the amount of materials required for its construction, which can be assumed proportional to the surface area. Using these, Equation 8 derives a cost-capacity relationship of vessels with different sizes. Given that radius of a sphere r is proportional to the cube root of its volume (V), Equation 9 can be derived, showing an economies of scale parameter of 2/3 or 0.67. Similar analytical approaches apply to other technologies, such as in.^39^^,^^74^ Finally, Equation 9 can be generalized into Equation 10, in which the capital cost C to build a unit of size $S_{t}$ can be estimated using the cost of a unit with size $S_{Ref}$. Here, parameter b is equal to 1 if the scale proportionally affects the cost, and less than 1 if larger units benefit from economies of scale. It is important to note, however, that economies of scale are not solely due to the physical factors. Other factors such as potential discounts from bulk material purchases, labor and capital efficiencies, increased manufacturing difficulties and materials strength requirements, also play roles.^75^ These factors can be difficult to separate from other factors. Hence, economies of scale parameters (b) in literature,^8^^,^^12^^,^^40^^,^^41^^,^^42^ including this work, are empirically estimated from historical data, rather than analytically calculated.

(Equation 8) $$\frac{C_{1}}{C_{2}}=\frac{4\pi {r_{1}}^{2}}{4\pi {r_{2}}^{2}}$$

(Equation 9) $$C_{1}=C_{2}{\left(\frac{V_{1}}{V_{2\;}}\right)}^{\frac{2}{3}}$$

(Equation 10) $$C_{t}=C_{Ref}\;{\left(\frac{S_{t}}{S_{Ref}}\right)}^{b}$$

Capital cost (C) is the product of the size of the technology and the specific investment cost (see Equation 11). By substituting C in Equation 10 with this definition, we derive Equation 12, which can then be simplified to Equations 13 and 14. As can be seen, Equation 13 demonstrates how the specific investment cost (IC) of a plant with different sizes can be estimated. Notably, this equation resembles the learning concept described in Equations 1 and 5. Therefore, drawing from the learning concept, we introduce the economies of scale rate (ESR), which explains the percentage reduction in cost with each doubling of the size of the unit or project, as shown in Equation 15.

(Equation 11) $$C_{t}=S_{t}\;IC_{t}$$

(Equation 12) $$S_{t}\;IC_{t}=S_{Ref}\;{IC}_{Ref}\;{\left(\frac{S_{t}}{S_{Ref}}\right)}^{b}$$

(Equation 13) $$IC_{t}={IC}_{Ref}\;{\left(\frac{S_{t}}{S_{Ref}}\right)}^{b-1}$$

(Equation 14) −β=b−1

(Equation 15) $$ESR=1-2^{-\beta }$$

**Figure 1:**
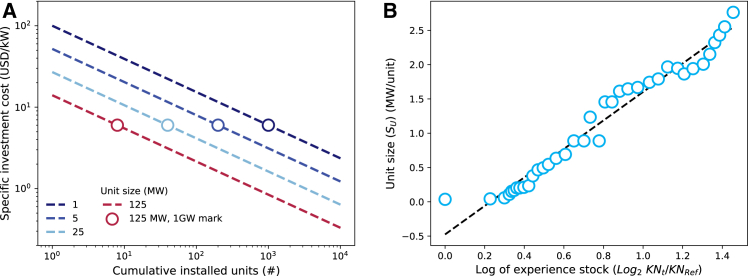
Illustrations of learning, economies of scale, and knowledge gap effects (A) Effects of learning and unit-level economies of scale on cost reductions. Solid lines represent cost trajectories for constant unit sizes, while dots indicate specific investment costs at 1 GW of cumulative capacity for each size-specific trajectory. (B) The knowledge gap effect dictates how quickly a technology can increase its size as a function of learning or experience.

The combination of the concepts above is illustrated in Figure 1A for a technology with equal learning and economies of scale rates. The dark blue line represents the cost trajectory if the technology development exclusively focused on learning through deploying a thousand 1 MW units to reach the 1GW mark, assuming an initial cost of $100/kW. Conversely, the red line depicts a strategy that initially emphasizes the economy of scale by increasing unit size to 125 MW to reduce the initial specific investment cost to $ 14/kW. In this strategy, further cost reduction to $6/kW is achieved through deploying 8 units of 125 MW each. The figure demonstrates that, due to similar learning and scaling rates, the same cost can be achieved at the 1 GW mark, regardless of the strategies. However, it is important to note that the learning effect decreases as unit size increases, which might be important for a technology with different learning and economies of scale effects.

Following the concepts illustrated in Figure 1A, prioritizing rapid upscaling is economically advantageous when economies of scale outweigh the learning effect in a technology. This applies to technologies where significant cost reductions through further experimentation are less likely due to a low learning rate or when the technology is approaching technical maturity. In such cases, rather than attempting to incrementally reduce costs via learning, size upscaling may provide significant cost reductions. However, changing the technology size too quickly might lead to dis-economies of scale, that adversely impact the technology cost.^39^^,^^76^ Numerous studies have provided statistical evidence as well as analytical and physical analyses of this phenomenon.^39^^,^^74^^,^^76^ Notwithstanding this, historical data analysis shows that the size at which dis-economies of scale starts to arise has increased over time.^39^^,^^44^ In this context, it is essential to recognize that increasing technology size also requires a learning process.^39^ As illustrated in Figure 1B for historical onshore wind data, the unit size can increase at a rate that is directly proportional to the logarithm of the experience stock. This relationship can be described by Equation 16.

As can be seen in Equation 16, γ represents size scale-up rate of a technology, with Smax indicating the maximum size the technology can achieve before the knowledge gap affects costs. In each period t, the technology size S can be smaller than, equal to, or greater than Smax. A positive variable δ in Equation 17 shows the technology size S relative to Smax. Here, auxiliary non-negative variables ${Cl}^{+}$ and ${Cl}^{-}$ are introduced in Equation 18. If S is greater than Smax, i.e., δ>1, ${Cl}^{-}$ is zero and ${Cl}^{+}$ is positive. Otherwise, ${Cl}^{-}$ is positive and ${Cl}^{+}$ is zero. In other words, ${Cl}^{+}$ represents fraction of S that exceeds Smax. Using Equations 16, 17, 18, and 19 can be derived.

(Equation 16) $${Smax}_{t}=S_{t-1}+\gamma \;\log_{2}\left(\frac{{KN}_{t}}{KN_{t-1}}\right)$$

(Equation 17) $$S_{t}=\delta_{t}\;{Smax}_{t}$$

(Equation 18) $$\delta =1+{Cl}^{+}-{Cl}^{-}$$

(Equation 19) $$\frac{S_{t}}{1+{Cl}^{+}-{Cl}^{-}}=S_{t-1}+\gamma \;\log_{2}\left(\frac{{KN}_{t}}{KN_{t-1}}\right)$$

In this work, the knowledge gap effect (KG) negates the gains from economies of scale (EoS) when technology size is increased exceeding Smax. This is shown in Equations 20 and 21. Accordingly, Equation 22 shows the knowledge gap effect as a function of S, Smax, and β for all S>Smax. As discussed earlier, this is the condition where δ is equal to $1+{Cl}^{+}$. Finally, Equation 23 is derived by substituting $1+{Cl}^{+}$ for δ in Equation 17 and then use the substitution result to replace S in Equation 22.

(Equation 20) $$1={KG}_{t}\;{EoS}_{t}\;\forall \;S_{t}>{Smax}_{t}$$

(Equation 21) $$1={KG}_{t}\;{\left(\frac{S_{t}}{{Smax}_{t}}\right)}^{-\beta }\;\forall \;S_{t}>{Smax}_{t}$$

(Equation 22) $${KG}_{t}={\left(\frac{S_{t}}{{Smax}_{t}}\right)}^{\beta }\;\forall \;S_{t}>{Smax}_{t}$$

(Equation 23) $${KG}_{t}={\left(1+{Cl}_{t}^{+}\right)}^{\beta }$$

By combining technology learning, economies of scale, and knowledge gap concepts at the unit and project levels, the specific investment cost can be described by Equations 24, 25, 26, 27, 28, 29, and 30. The A term reflects the learning effect, while the third and fourth blocks of Equation 25 (Bu and Bp) represent the economies of scale effect. Additionally, the $1+{Cu}^{+}$ and $1+{Cp}^{+}$ terms capture the knowledge gap effect, with $\gamma_{u}$ and $\gamma_{p}$ as scale-up rate parameters at unit and project levels, respectively. Cu and Cp here are similar to Cl in the illustration used in Equations 18 and 19.

(Equation 24) $$IC_{t}={IC}_{Ref}\times {Learning}_{t}\times {EoS}_{unit,t}\times {EoS}_{project,t}\times {KG}_{unit,t}\times {KG}_{project,t}$$

(Equation 25) $$IC_{t}={IC}_{Ref}\;{A_{t}}^{-\alpha }\;{{Bu}_{t}}^{{-\beta }_{u}}\;{{Bp}_{t}}^{{-\beta }_{p}}\;{\left(1+{Cu}_{t}^{+}\right)}^{\beta_{u}}\;{\left(1+{Cp}_{t}^{+}\right)}^{\beta_{p}}$$

(Equation 26) $$A_{t}=\frac{KN_{t}}{{KN}_{Ref}}$$

(Equation 27) $${Bu}_{t}=\frac{{Su}_{t}}{{Su}_{Ref}}$$

(Equation 28) $${Bp}_{t}=\frac{{Sp}_{t}}{{Sp}_{Ref}}$$

(Equation 29) $$\frac{{Su}_{t}}{1+{Cu}_{t}^{+}-{Cu}_{t}^{-}}={Su}_{t-1}+\gamma_{u}\;\log_{2}\left(\frac{{KN}_{t}}{KN_{t-1}}\right)$$

(Equation 30) $$\frac{{Sp}_{n}}{1+{Cp}_{t}^{+}-{Cp}_{t}^{-}}={Sp}_{t-1}+\gamma_{p}\;\log_{2}\left(\frac{{KN}_{t}}{KN_{t-1}}\right)$$

As mentioned earlier, this study aims to quantify the roles of factors affecting investment cost reductions in technologies, focusing on learning and economies of scale at both unit and project levels. Additionally, it provides a quantitative analysis of the knowledge gap effect, which measures how quickly unit and project sizes can be increased with a given addition of experience stock. It is also aimed to provide the required data and parameters to perform the analysis and implement the concept in energy-economy models.

To achieve these goals, we used the historical timeseries data of investment cost, cumulative installed capacity, and sizes at unit and project levels. First, we used the data to estimate the scale-up rate parameters (γ), which measure the rates of unit and project sizes increase for a given experience stock addition. Here, unit and project sizes are expressed in capacity per unit and number of units per project, respectively, to avoid economies of scale at unit level being attributed to the project level. Following this, we used these parameters to identify how much the scale up rates historically exceed the “knowledge limit” for each period ($Cu^{+}$ and $Cp^{+}$). For these given parameters, learning (α) and economies of scale parameters ($\beta_{u}$ and $\beta_{p}$) can be estimated.

Following this, we use the estimated parameters to quantify the roles of each factor and compare historical data with curve fits from the proposed approach and the conventional one-factor learning. In this context, comparison with other multifactor learning approaches might be valuable. However, this work does not pursue this analysis due to the limited availability of data and parameters necessary for such comparisons across the technologies examined. Finally, we provide a tool that can be used to implement this approach in energy economy models. Using installed capacity projections from the literature, we used the tool to estimate future costs of several power generation technologies, serving as a proof of concept for our proposed methods. We then demonstrate how the results compare with other estimates in the literature.

In this study, we focus on economies of scale effects at the unit or project levels of the technology, such as the impacts of wind turbines rated capacity and PV project sizes on costs. Economies of scale at higher levels, such as supply chain and manufacturing facilities^7^^,^^8^^,^^44^^,^^77^^,^^78^ are not within the scope of this work. Therefore, their effects remain aggregated in the overall learning effect. It is also important to note that in addition to these supply chain and manufacturing economies of scales, other factors we mentioned earlier in the introduction can also substantially contribute to technology cost changes. Previous studies^8^^,^^9^^,^^10^^,^^11^^,^^12^^,^^13^^,^^16^^,^^47^^,^^48^^,^^49^ have contributed to disentangle those effects. Rather than developing a new approach to entirely replace them, this approach complements those contributions by providing the research community and policymakers a new tool and data to disentangle economies of scale effects at the unit and project levels. Therefore, this contribution can be integrated to enhance factors represented in the multi-factor learning concept. Particularly, our contribution allows quantification of the optimal rates at which technology sizes can be scaled up.

## Results and discussion

### Roles of different factors in technology cost changes

Using the parameter estimation model, we quantify the role of each factor affecting cost changes in power generation technologies, namely learning, economies of scale, and knowledge gap at unit and plant levels.

**Figure 2:**
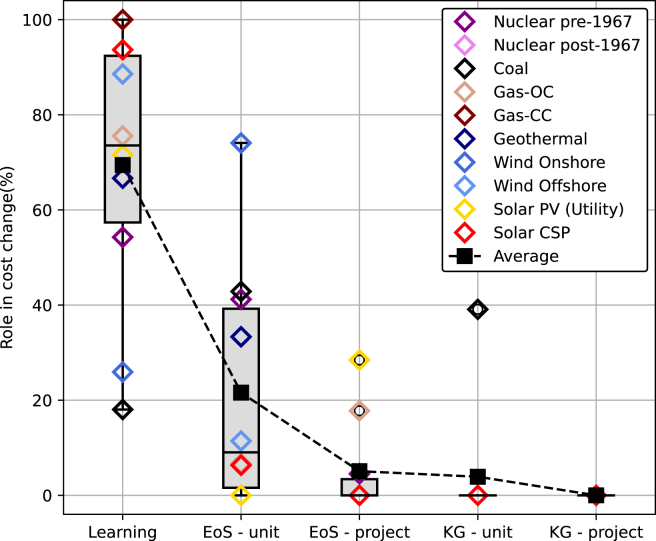
Roles of different factors in historical cost changes across power generation technologies Using the proposed approach, we quantify the roles of learning, economies of scale (EoS), and knowledge gap (KG) effects at both the unit and project levels. This figure illustrates the contributions of these factors to total historical cost changes in absolute terms, with the total for each technology adding up to 100%. Data are represented as median with interquartile range (IQR).

As shown in Figure 2, the learning effect dominates cost changes, averaging around 69% of the total. Following the learning effect, economies of scale at unit and project levels on average drive 22% and 5% of the changes, respectively. Interestingly, the impact of cost penalty due to rapid changes in unit and project sizes to the total change tends to be limited as technologies gradually scale up their sizes. Notwithstanding this, for some technologies such as coal power plants, this knowledge gap factor at the unit level can be essential.

The learning effect plays a significant role in technologies with limited scalability, particularly those at early development stages, such as concentrated solar power (CSP), which undergo initial experimentation and demonstration at smaller scales. This effect is also observed in mature technologies, such as Nuclear post-1967, Gas-CC, and Gas-OC, which have reached their maximum size. While the impact on costs is negligible for the latter group, our results demonstrate a 59% reduction in the cost of CSP as experience accumulates rapidly.

As scalable technologies progress and mature over time, the role of learning in changing the costs diminishes. Our results show that technological learning in coal, onshore wind, and solar PV, for instance, contributes to around 18–71% of cost changes. For those technologies, further cost reduction can be achieved by increasing the size of the technology at the unit (e.g., wind and coal) and project (e.g., solar PV) levels. For wind, the economies of scale at the unit level account for 74% of technology cost changes. To achieve this, the unit sizes of onshore wind were increased by 100 times the initial sizes. On the other hand, the impact of economies of scale at the project level was significant for solar PV. Hence, further cost reductions were achieved by increasing the number of units at the same project. Interestingly, project sizes marginally affect the cost of wind due to the minor contribution of expenses at project level to the total investment cost.

In economies of scale, the plant’s cost structure plays a crucial role. For PV, the impact of economies of scale at the project level is notably stronger than that at the unit level. In this context, module prices constitute only 18–50% of the technology cost.^79^ In other words, around 50–82% of costs arise from the shareable balance of system and infrastructure expenditures, allowing the technologies to considerably reduce costs via increasing project sizes. Conversely, for wind, 80% of the costs are spent at the unit level, i.e., for turbine, foundation, and assembly (installation), with only 13% of the total for shareable items, such as site access, electrical infrastructure, engineering, and development.^80^ In addition, the turbines for the same project are installed far apart, further limiting cost reduction potential through shared infrastructure. As a result, efforts to reduce cost at the unit level, such as via increasing turbine size, are a priority for wind technologies.

While increasing the unit size can result in cost reductions, rapid scaling may incur a cost penalty due to knowledge gap. For example, a swift increase in the size of Coal contributes to a 39% cost penalty. As technologies diverge significantly from prior experiences, a knowledge gap effect emerges, negating the benefits derived from previous learning. Our results demonstrate that this effect is noticeable at the unit level but more manageable at the project level. As increasing unit size may require new technology and supply chain setup, increasing project size can be achieved simply by increasing the number of units. Thus, as shown in Figure 2, the knowledge gap effect at the project level is negligible for all technologies.

### Separated learning and economies of scale can explain cost dynamics better

**Figure 3:**
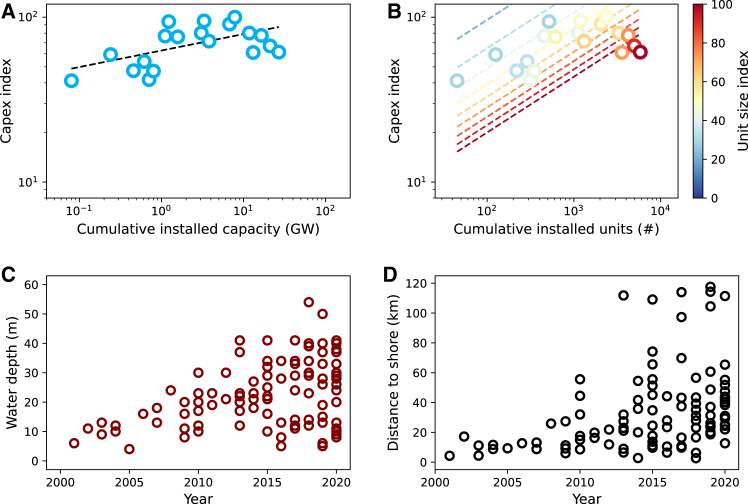
Historical data and curve fit for offshore wind (A–D) These figures illustrate insights from (A) one-factor learning and (B) separated learning and economies of scale approaches. Figure A shows that offshore wind deployment slightly increases technology costs due to a negative learning rate. In contrast, Figure B demonstrates that, although increasing the number of units increases costs, unit size scale-up can significantly reduce costs. Historical (C) water depth and (D) distance to shore data of offshore wind projects illustrate how other factors can affect the aggregated learning value of a technology. Data for (C) and (D) are adopted from Musial.^81^

We showed that economies of scale can significantly contribute to cost reductions. As a result, separating learning and economies of scale can explain technology cost dynamics better than the approach that exclusively uses the learning concept. To illustrate, Figure 3 compares historical data fit between one-factor learning and the proposed approach, that is the separated learning and economies of scale approach, for offshore wind. As can be seen in Figure 3A, the one-factor learning approach shows that the potential for offshore wind cost reductions is negligible. Doubling the technology capacity slightly increases the cost rather than decreasing it. This result is in line with the values of the learning rates reported by other studies. In this context, some studies prefer to also report segmented values of the learning rates, showing negative learning for the first segment and positive learning afterward.^35^

Figure 3B demonstrates that the separated learning and economies of scale approach can distinguish the impact of each factor on the technology cost dynamics. Using this approach, we show that doubling the number of units increases the cost of offshore wind by 28%. In addition, the approach also identifies that the technology can significantly benefit from increasing the unit size. As can be seen in the Figure, doubling the unit size reduces the cost of offshore wind by 48%. This explains why up to a thousand units of turbines are installed, that is a period with constant unit size, the cost of offshore wind increases, followed by rapid cost reductions as turbine sizes increase. Further investigation from the literature shows that the negative learning of offshore wind is, among other factors, strongly caused by an increased sea depth and distance from the shore at which recent turbines were installed.^11^^,^^82^ As the current global offshore wind markets are dominated by China, the UK, and Germany, increasing the number of units rapidly occupying potential sites with shallow sea floors near the shore. Such a factor explains why area/site-specific technologies, such as offshore wind and geothermal, may have negative learning rates (see Tables 2 and 3). As seen in Figure 3C and 3D, the depth and distance to the shore of offshore wind rapidly increase.

**Table 2:** Technology change parameters for separated learning and economies of scale approach

Technology	${IC}_{Ref}$, $/kW	LR, %	ESR (unit), %	ESR (project), %	SUR(γ) (unit), MW	SUR(γ) (project), # of Units
Nuclear pre-1967	4699	−11.7	42.6	38.4	338.5	0.89
Nuclear post-1967	1719	−17.3	0.0	0.0	576.4	0.59
Coal	2131	14.1	13.5	0.0	1442.3	1.14
Gas-OC	946	3.4	2.1	9.2	N/A^a^	1.05
Gas-CC	1055	1.4	0.0	0.0	224.9	N/A^a^
Geothermal	3560	−100.0	21.0	0.0	67.5	3.39
Onshore Wind	5005	4.7	13.5	0.0	2.2	N/A^a^
Offshore Wind	2313	−27.5	47.5	0.0	2.1	N/A^a^
Solar PV (Utility)	5690	22.1	N/A^b^	21.5	N/A^b^	108,401.72^c^
Solar CSP	27356	18.8	2.7	19.9	68.0	N/A^a^

${IC}_{Ref}$, investment cost reference; LR, learning rate; ESR, economies of scale rate; SUR, scale up rate (γ).

a Not applicable. Unit/project size trends show constant or decreasing.

b Not applicable. Unit size for Solar-PV is assumed constant at 300 W/panel.

c The SUR at the project level for solar PV is high compared to other technologies due to the significantly smaller unit size of this technology compared to others.

**Table 3:** Technology change parameters for one-factor learning approach

Technology	${IC}_{Ref}$, $/kW	LR, %
Nuclear pre-1967	14760	21.1
Nuclear post-1967	1733	−14.7
Coal	1677	7.6
Gas-OC	1062	6.0
Gas-CC	1051	1.0
Geothermal	2923	−96.0
Onshore Wind	3967	8.9
Offshore Wind	2678	−6.8
Solar PV (Utility)	6330	27.3
Solar CSP	22205	14.6

${IC}_{Ref}$, investment cost reference; LR, learning rate.

**Table 4:** Technology data sources

Technology	Data Sources
Nuclear	Lovering et al.^85^; IAEA^87^
Coal	McNerney et al.^88^; Yeh & Rubin^89^
Gas-CC	Colpier & Cornland^90^; International Energy Agency^91^
Gas-OC	Rogner^92^
Geothermal	IRENA^79^; IRENA^83^; Ediger & Akar^93^; Barbier^94^; International Energy Agency^95^
Onshore Wind	IRENA^79^; IRENA^83^; International Energy Agency^95^; ^96^; Wiser et al.^97^
Offshore Wind	IRENA^79^; IRENA^83^; International Energy Agency^95^
Solar PV (Utility)	IRENA^79^; IRENA^83^; International Energy Agency^95^; Barbose et al.^98^; Barbose & Darghouth^99^
Solar CSP	IRENA^79^; IRENA^83^; International Energy Agency^95^; Lilliestam et al.^100^; Thonig et al.^101^

**Figure 4:**
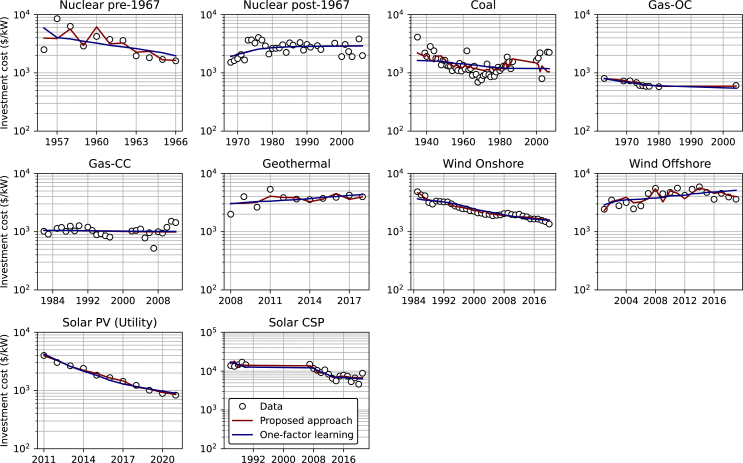
Comparison between historical data, one-factor learning, and the proposed approach for different technologies

Finally, we compare historical data with curve fits based on one-factor learning and the proposed approach for power generation technologies. As shown in Figure 4, the proposed approach consistently outperforms the one-factor learning in explaining the cost dynamics of power generation technologies, especially for technologies affected by economies of scale at unit or project levels.

### Technology cost change parameters

This subsection discusses cost change parameters associated with power generation technologies. Parameters for separated learning and economies of scale approach are summarized in Table 2. The parameters for one-factor learning are presented in Table 3. The historical data used to derive these parameters are from sources outlined in Table 4, with details provided in Figure 7 in the STAR Methods and Tables S1–S10 in the supplemental information.

Table 2 illustrates the varying effects of each factor on potential cost reduction across technologies. Cost reduction for onshore wind, for example, is strongly dominated by scaling up the unit size. Here, doubling the size reduces costs by 13.5%, almost triple the effect of learning. Thus, increasing the size of the turbines is a priority in cost-reduction efforts for onshore wind. Conversely, for coal, the learning effect is comparable to the impact of economies of scale at the unit level. This highlights the need to balance the speed of installing more units and increasing the unit size to accelerate technology cost reduction. Interestingly, both learning and economies of scale at the project level strongly and positively affect cost reduction in Solar PV, allowing the technology to reduce its cost by more than 80% in the last decade alone.^83^

In the one-factor learning approach, cost changes solely depend on learning rates, making it easy to identify the direction of cost change for the investment cost of newly installed capacity. As shown in Table 3, while the investment cost of the newly installed capacity of most technologies is expected to be cheaper, the increased costs of Nuclear post-1967, geothermal, and offshore wind should be anticipated. Although the approach is useful for estimating the direction and magnitude of technology cost change, as discussed in the motivation of this study, it cannot be used to identify strategies to accelerate cost reduction. Most importantly, aggregating all factors affecting cost change into a single predictor, i.e., cumulative installed capacity, can be misleading. In this context, the approach simply assumes that the historical trends will persist in the future, which may not be the case. For instance, the upper limit for technology unit size, dictated by construction structure, material strength, supply chain, or other factors, constrains the application of one-factor learning. This limitation can lead to an overestimation of cost reduction for technologies exhibiting a strong economy of scale effect that is already approaching its unit size limit.

**Figure 5:**
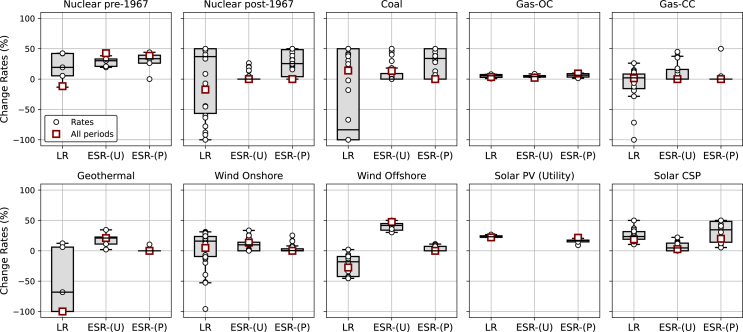
Technology change parameters across different historical time segments compared to full dataset estimates Each plot displays the ranges of learning (LR) and economies of scale (ESR) rates for various power generation technologies at the unit (U) and project (P) levels. We assumed constant panel size for PV, therefore, ESR(U) plot for the technology is left empty. Black circle dots represent change rates across different historical time segments, with boxplots illustrating their range. Maroon rectangular dots indicate the rates estimated using the full dataset. Positive values in this figure indicate the percentage of cost reductions that can be achieved by doubling the cumulative number of units and sizes for each respective factor. The figure also highlights that some technologies show both positive and negative learning rates across different periods, driven by a range of factors, such as regulatory and site/resource constraints during the corresponding periods, underscoring the importance of disentangling these factors from learning effects to better understand cost dynamics. Data are represented as median with interquartile range (IQR).

Technology innovation is a complex process where uncertainty is inherent and can significantly affect the quality of future cost estimates. While the values above show parameters estimated across the full period data we have, here, we also show the distribution of those parameters across different time segments. As can be observed in Figure 5, parameters associated with economies of scale exhibit robustness, while those related to the learning effect may have a higher level of uncertainty. For economies of scale, the robustness of the parameter can be attributed to physical factors, such as materials and installation cost savings, that are less affected by temporal factors. On the other hand, the learning effect may be affected by safety and environmental regulations, currency variations, material prices, and other factors that consistently evolve. This result highlights the need to regularly update historical data to allow policy designs derived from learning assumptions to adapt to more recent observations. In addition, it also emphasizes the significance of considering multiple factors influencing technology cost change rather than solely relying on the one-factor learning approach.

### Future outlook of technology costs

In this section, we used the IEA’s installed capacity projections to estimate future technology costs using our proposed tool, which optimizes the number of units and technology sizes at both the unit and project levels. We compared our cost projections with those from the IEA. While changes in technology costs can influence future capacity projections, energy prices, and demand in energy-economy models that implement this tool, exploring these interactions is beyond the scope of our study. This section demonstrates a proof of concept for our methods in quantifying cost reductions from learning and economies of scale effects while endogenously limiting unit and project size upscaling through the knowledge gap effect.

**Figure 6:**
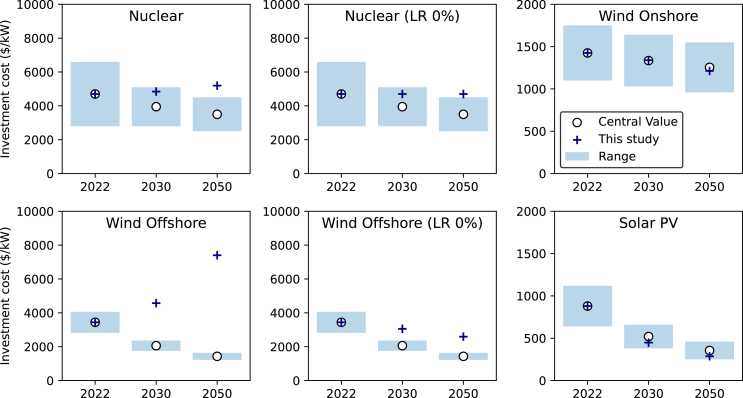
Projected investment cost benchmark This study uses future capacity and the range of investment costs from the IEA.^84^ Estimates were performed using the current central value of investment costs as the reference. This figure shows that our projections for Wind Onshore and Solar PV align with the IEA’s estimates. However, our results for Nuclear and Wind Offshore diverge from the IEA’s projections due to assumed negative learning rates, which were primarily driven by increased safety requirements for Nuclear and greater water depths at deployment sites for Wind Offshore. Additionally, we provide projections using a 0% learning rate to illustrate the future costs of these technologies if the negative learning effect is disregarded. Similar analyses for Wind Onshore and Solar PV were not performed as these technologies face relatively fewer regulatory and site constraints that can significantly affect their observed learning rates.

Figure 6 compares investment cost projections between our proposed approach and existing literature. We derived future costs for wind, solar PV, and nuclear technologies using central values from the literature for current investment cost and future capacity estimates, along with technology cost change parameters from Tables 2 and 3. Other technologies are excluded from the comparison due to unavailable cost or future capacity estimates in the literature.^84^

**Figure 7:**
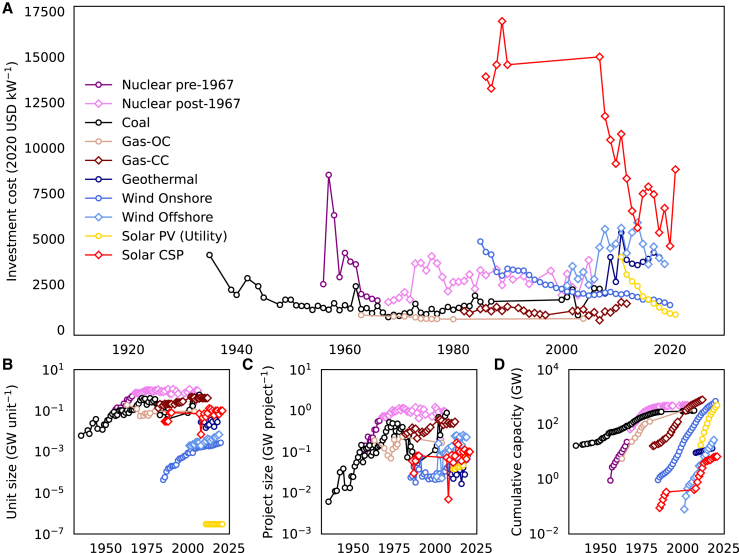
Historical data for parameter estimation in the proposed approach (A) investment cost, (B) annual average unit size, (C) annual average project size, and (D) cumulative installed capacity of power generation technologies.

Our projections for offshore wind and nuclear diverge from the IEA estimates. As can be seen in the figure, the IEA assumed that nuclear will be 11–32% cheaper than the current levels. Our study finds that the cost of nuclear increases by 23% every doubling the number of units while the potential for economies of scale is limited as the technology is already at a GW scale per project and may not be easily increased further. Similarly, our study also found a negative learning rate for offshore wind.

As discussed earlier, negative learning rates for offshore wind is mainly due to the increased water depth at deployment sites in the countries currently deploying the technology.^11^^,^^82^ This trend might change if the technology is adopted in other regions where sites with shallower water are available. On the other hand, negative learning rates for nuclear is contributed by the required improvements in safety performance that leads to an increase in system complexity for the technology construction.^85^ As an exercise to these, we estimated potential cost reductions for nuclear and offshore wind by omitting the learning effect, focusing solely on the impact of economies of scale.

If the negative learning effect is neglected, our results indicate a potential 25% reduction in offshore wind investment costs by 2050. These reductions can be achieved by increasing the unit size of offshore wind from the current global average of 7.7 MW/unit to 8.7 MW/unit by 2030 and 10.4 MW/unit by 2050. For nuclear, the impact of economies of scale on future costs is negligible as the technology is approaching its maximum size. While onshore wind capacity is projected to increase 8-fold,^84^ our estimates show that the cost is expected to marginally decrease from $1425/kW to $1213/kW by 2050. This modest reduction is attributed to the unit size reaching 4.2 MW/unit, approximately 50% larger than the current size of 3.0 MW/unit, with only a moderate economies of scale effect. It is important to note that the project-level economies of scale for Wind technologies are negligible.

The cost of solar PV is also expected to rapidly decrease from currently $880/kW to $287/kW by 2050. Here, cost reductions are attributed to both learning and project-level economies of scale. In this context, cost reduction is accelerated by rapidly increasing project size, allowing the technology to benefit from the project-level economies of scale without affecting cost reduction from the learning effect. As a result, the cost estimates for solar PV in our study are slightly more optimistic than the benchmark value.

### Conclusion

In this study, we demonstrated that learning, economies of scale, and knowledge gap at the unit and project levels can significantly affect the costs of power generation technologies. Thus, our paper offers methodologies and parameter data crucial for technology cost estimation by considering these factors.

Notably, we identified limitations in the common practice of using aggregated one-factor learning to estimate future technology costs. This approach’s assumption that historical unit and project size scale-up trends will persist in the future can be misleading. It may underestimate cost declines during upscaling periods for technologies with strong economies of scale effects and overestimate cost reductions for technologies that have reached their maximum size.

Most importantly, we observe that enhancing the factors accounted for in cost estimations for several technologies shows significantly lower learning rates than the rates observed in the one-factor learning approach. Estimates developed using our approach better fit the historical data than the one-factor learning curve. These findings suggest that a substantial portion of the historical cost decline in these technologies is attributable to economies of scale rather than learning processes.

Moreover, our newly introduced knowledge gap factor can capture trade-offs between rapid scale-up of unit size, to quickly harness benefits from economies of scale, and the knowledge gap effect that negates benefits from previous learning. By offering a more comprehensive understanding of these dynamics, our proposed approach allows decision-makers to design more informed choices in technology development to accelerate technology cost reductions and deployment.

In light of this conclusion, while the utilization of one-factor learning within IAMs (and other energy economy models in general) is able to capture the cost dynamics of emerging yet fairly established technologies, its continued application may inherently underestimate the timing for novel technologies to achieve cost competitiveness and the magnitude of their role. This is particularly likely to be observed in those technologies with strong potentials for harnessing economies of scale effect, but still in the nascent developmental phase. This primarily arises from the small-scale deployment of technology at this stage for demonstration and experimentation purposes, thus impeding its ability to fully leverage the potential cost reductions stemming from economies of scale. Notably, the one-factor learning approach inherently assumes that these trends persist, implying that the technology will never be able to harness the economy of scale. This behavior is highlighted in^29^ which shows that IAMs’ forecasts often underestimate the cost reduction of renewables and batteries in the early phase of their development. Additionally, the competitiveness of such technologies is further underestimated, as the approach assumes that mature technologies still have significant room for improvement (i.e., unit size scale-up) even though they may have already reached their maximum size, such as In the case of nuclear.^29^^,^^86^

### Limitations of the study

Although the technological learning concept is valuable for estimating future costs, it has faced significant criticism for its heavy reliance on cumulative installed capacity as explanatory variables.^7^ This study attempted to fill this gap by providing tools to separate economies of scale at the unit or project levels from learning effects, along with data and parameters to estimate future technology costs using the proposed concept. We also introduce a knowledge gap effect to quantify how quickly a technology can scale up its size to harness the economies of scale effect.

In addition to these contributions, this study identifies several limitations and areas for future research that warrant attention and would enhance the insights obtained thus far.

Firstly, despite its significance in disentangling economies of scale at the unit and project levels, the learning effect in this study remains an aggregate of other factors. These include the “true” learning, regulations, resource depletions, R&D expenditures, knowledge spillover, and economies of scale at higher levels (e.g., supply chain and manufacturing facilities), etc. Fully separating these factors requires additional data, which may be difficult to obtain and likely to vary in quality, regional coverage, and increasingly more complex system boundaries.^7^ Nevertheless, these factors can significantly contribute to cost changes and may, in some cases, also require to be disaggregated from learning. For instance, in line with our findings at the unit and project levels, aggregating the economies of scale at the supply chain and manufacturing facilities levels into the “overall” learning effect can further underestimate cost reductions potential for granular and modular technologies.^7^^,^^44^ This is particularly relevant, given the weak correlation between the learning effect of the technology and the scales of supply chain and manufacturing facilities.^7^ Therefore, this work complements existing efforts to disentangle factors contributing to cost changes, offering the research community additional tools to enhance dynamic representations of technology costs in energy-economy models.

Secondly, this study focuses on the capital cost of technology. Hence, the effects of learning, economies of scale, and knowledge gap on other technology features, such as efficiency, lifetime, capacity factor, emissions factor, etc., are not captured. Notwithstanding this, the application of the proposed approach in energy-economy models can be easily expanded to include other technological features to allow the approach capturing technology progress more holistically.

Finally, in conducting the analysis, we used data from various sources which may have different levels of completeness. This may lead to data uncertainty issues that can significantly affect the results and analysis.^7^ Although this limitation is inherent in modeling work, this emphasizes the importance of continuously updating data input when using this approach for decision-making processes.

## Resource availability

### Lead contact

Further information and requests for resources should be directed to and will be fulfilled by the lead contact, Yoga W. Pratama (pratama@iiasa.ac.at).

### Materials availability

This study did not generate new materials.

### Data and code availability

• All data reported in this paper are available in the supplemental information.
• All original code used in this paper is available in the supplemental information.
• Any additional information required to reanalyze the data reported in this paper is available from the lead contact upon request.

## Acknowledgments

The authors acknowledge and appreciate funding under the 10.13039/100011102European Union’s ERC-2020-SyG ‘GENIE’ Grant, Grant ID 951542.

## Author contributions

Conceptualization: Y.W.P. and M.J.G.; Methodology: Y.W.P. and M.J.G.; Investigation: Y.W.P.; Visualization: Y.W.P.; Funding acquisition: K.R.; Supervision: M.J.G.; Writing – original draft: Y.W.P.; Writing – review and editing: Y.W.P., M.J.G., J.G., A.Z., G.N., and K.R..

## Declaration of interests

The authors declare no competing interests.

## STAR★Methods

### Key resources table

**Table:** 

REAGENT or RESOURCE	SOURCE	IDENTIFIER
**Deposited data**		

Nuclear pre-1967 Historical Data	This paper	Table S1
Nuclear post-1967 Historical Data	This paper	Table S2
Coal Historical Data	This paper	Table S3
Gas OC (Open Cycle) Historical Data	This paper	Table S4
Gas CC (Combined Cycle) Historical Data	This paper	Table S5
Geothermal Historical Data	This paper	Table S6
Wind Onshore Historical Data	This paper	Table S7
Wind Offshore Historical Data	This paper	Table S8
Solar PV (Utility) Historical Data	This paper	Table S9
Solar CSP Historical Data	This paper	Table S10
Wind Offshore Depth Historical Data	This paper	Table S11
Wind Offshore Distance to Shore Historical Data	This paper	Table S12

**Software and algorithms**		

Code to perform the analysis	Zenodo	Zenodo: https://doi.org/10.5281/zenodo.14387258

### Method details

#### Data collection

In this work, the historical data on cumulative installed capacity, the number and average size of units and projects, as well as the investment costs are required to estimate learning and economies of scale parameters. For each technology, data sources used for this study are listed in Table 4.

It is important to note that some assumptions need to be made when the required data are not available. Among those data, the number of units and unit size are not always available. If either of these is available, the other can be estimated using capacity addition data. If both data are not available, we used Global Power Tracker^102^^,^^103^^,^^104^ data, which list installed units, projects, and their sizes, to estimate the annual average unit and project sizes of the technology for each period. Figure 7 below visualizes the data input used for parameter estimation for the various technologies included in this study. The data and code required to perform the analysis for this paper can be seen in the supplemental data and code.

#### Parameter estimation model

While one-factor learning parameters for numerous technologies have been well documented, literature on the parameters for separated learning and economies of scale is limited.^8^^,^^42^^,^^45^^,^^105^ In this work, we used Equation 25 and the curve-fitting approach to estimate the learning (α) and economies of scale (β) parameters, as well as the scale up rate (γ) parameter to incorporate knowledge gap effect. To perform this, we used the number and size of installed units and projects, as well as investment cost historical data, as an input for the parameter estimation model, aiming to minimize the least square error. Here, Equation 25 is linearized via the log-log transformation, shown in Equation 31, allowing the model becomes more tractable.

(Equation 31) $$\log_{2}\;IC_{t}=\log_{2}{IC}_{Ref}-\alpha \;\log_{2}A_{t}-\beta_{u}\;\log_{2}{Bu}_{t}-\beta_{p}\;\log_{2}{Bp}_{t}+\beta_{u}\;\log_{2}\left(1+{Cu}_{t}\right)+\beta_{p}\;\log_{2}\left(1+{Cp}_{t}\right)$$

Therefore, the objective function of the model is:

(Equation 32) $$\mathrm{Min}\;._{\alpha ,\beta }=\sum_{t}^{T}x_{t}^{2}$$

where $x_{t}$ is the estimation error (residual) in period t, that is the distance between the estimated and historical values of $\mathrm{Log}\;IC_{t}$, calculated using Equation 33. ${ic}_{Ref}$ in this equation is the intercept of the fitted curve with the y axis, which implies the estimated value of the reference plant’s specific investment cost. Equations 29 and 30 is included as constraints in its logarithmic form, shown in Equations 34 and 35.

(Equation 33) $$x_{t}=\log_{2}\;{ic}_{t}-\log_{2}{IC}_{Ref}+\alpha \;\log_{2}A_{t}+\beta_{u}\;\log_{2}{Bu}_{t}+\beta_{p}\;\log_{2}{Bp}_{t}-\beta_{u}\;\sum_{t^{\prime }}^{t}\log_{2}\left(1+{cu}_{t^{\prime }}\right)-\beta_{p}\sum_{t^{\prime }}^{t}\log_{2}\left(1+{cp}_{t^{\prime }}\right)\;\forall \;t^{\prime }\leq t$$

(Equation 34) $$\frac{{su}_{t}}{1+{cu}_{t}}\leq {su}_{t-1}+\gamma_{u}\;\log_{2}\left(\frac{{kn}_{t}}{{kn}_{t-1}}\right)$$

(Equation 35) $$\frac{{sp}_{t}}{1+{cp}_{t}}\leq {sp}_{t-1}+\gamma_{p}\;\log_{2}\left(\frac{{kn}_{t}}{{kn}_{t-1}}\right)$$

To estimate learning parameters for the one-factor learning, Equation 31 is replaced by Equation 36, which is derived from the linearized form of Equation 1. Hence, estimation residuals are calculated by using Equation 37. For one factor learning, Equations 34 and 35 are omitted.

(Equation 36) $$\log_{2}{ic}_{t}=\log_{2}{IC}_{Ref}-\alpha \;\log_{2}\left(\frac{{kq}_{t}}{{kq}_{Ref}}\right)$$

(Equation 37) $$x_{t}=\log_{2}{ic}_{t}-\log_{2}{IC}_{Ref}+\alpha \;\log_{2}\left(\frac{{kq}_{t}}{{kq}_{Ref}}\right)$$

(Equation 38) $${IC}_{Ref},\beta_{u},\beta_{p},\gamma_{u},\gamma_{p}\geq 0$$

#### Cost estimation model

One of the advantages of separating learning and economies of scale effects is that the trade-offs between the two effects can be quantified to optimize technology cost improvement. Here, we introduce an optimization model to perform this type of analysis. As shown In Equation 39, the objective of the model is to minimize total investment cost by varying the number and size at the unit and project levels, as well as the speed of unit scale up. By minimizing total investment cost, this implies in the model that cost reduction is capacity weighted. As a result, the model may select strategies that initially focus on learning to slowly reduce cost during the formative phase and increase unit and project sizes later to reduce cost further when the technology is rapidly deployed. Strategies might be different if the focus is rapid cost reduction during the formative phase to gain market share, which might be preferable from technology developers' perspective.

(Equation 39) $$\mathrm{Min}\;._{N,KN,Su,Sp}=\sum_{t}^{T}{INV}_{t}$$

Here, annual investment (INV) is a function of specific investment cost and capacity deployment in each period. Although future capacity is assumed as a parameter, the number of units and unit sizes are decision variables (Equation 40) which, in turn, can affect specific investment cost (Equations 41 and 42).

(Equation 40) $${INV}_{t}={IC}_{t}\;q_{t}={IC}_{t}\;N_{t}\;S_{t}$$

(Equation 41) $$IC_{t}={IC}_{t-1}\;{\left(\frac{{KN}_{t}}{KN_{t-1}}\right)}^{-\alpha }{\left(\frac{{Su}_{t}}{{Su}_{t-1}}\right)}^{-\beta_{u}}{\left(\frac{{Sp}_{t}}{{Sp}_{t-1}}\right)}^{-\beta_{p}}{\left(1+{Cu}_{t}\right)}^{\beta_{u}}\;{\left(1+{Cp}_{t}\right)}^{\beta_{p}}$$

(Equation 42) $${KN}_{t}={KN}_{t-1}+N_{t}$$

Similar to the parameter estimation model, this problem is solved in its logarithmic form for tractability. The log-transformed equivalent of the cost estimation problem is outlined in Equations 43, 44, 45, and 46.

(Equation 43) $$\min_{N,KN,Su,Sp}\sum_{t}^{T}2^{\log_{2}\;{INV}_{t}}$$

(Equation 44) $$\log_{2}\left({INV}_{t}\right)=\log_{2}\left({IC}_{t}\right)+\log_{2}\left(N_{t}\right)+\log_{2}\left(S_{t}\right)$$

(Equation 45) $$\log_{2}\left(IC_{t}\right)=\log_{2}\left({IC}_{t-1}\right)-\alpha \;\log_{2}\left(A_{t}\right)-\beta_{u}\;\log_{2}\left({Bu}_{t}\right)-\beta_{p}\;\log_{2}\left({Bp}_{t}\right)+\beta_{u}\;\log_{2}\left(1+{Cu}_{t}\right)+\beta_{p}\;\log_{2}\left(1+{Cp}_{t}\right)$$

(Equation 46) $${KN}_{t}={KN}_{t-1}+N_{t}$$

In this model, variable ${Cu}_{t}$ and ${Cp}_{t}$ represent the acceleration of unit and project scale up rates required to exceed unit size achievable by the technology at the normal scale up rate, calculated using Equations 47 and 48, respectively.

(Equation 47) $$\frac{{Su}_{t}}{1+{Cu}_{t}}\leq {Su}_{t-1}+\gamma_{u}\;\log_{2}\left(\frac{{KN}_{t}}{KN_{t-1}}\right)$$

(Equation 48) $$\frac{{Sp}_{t}}{1+{Cp}_{t}}\leq {Sp}_{t-1}+\gamma_{p}\;\log_{2}\left(\frac{{KN}_{t}}{KN_{t-1}}\right)$$

Meanwhile, cost estimates using one factor learning approach are preformed using Equations 49 and 50.

(Equation 49) $$\log_{2}\left(IC_{t}\right)=\log_{2}\left({IC}_{Ref}\right)-\beta \;\log_{2}\left(\frac{{kq}_{t}}{{kq}_{Ref}}\right)$$

(Equation 50) $${kq}_{t}={kq}_{t-1}+q_{t}$$

### Quantification and statistical analysis

All data expressed as median with interquartile range are indicated on the appropriate figure legends, as appears in Figures 2 and 5. Numpy Python package was used for these statistical analysis. Matplotlib Python package was used to generate all the figures.

## Footnotes

Supplemental information can be found online at https://doi.org/10.1016/j.isci.2024.111644.
