# Supplementary material for: Learning, economies of scale, and knowledge gap effects on power generation technology cost improvements
Source: iScience. 2024 Dec 19;28(1):111644. doi: 10.1016/j.isci.2024.111644 (PMC11761306; doi:10.1016/j.isci.2024.111644)
Supplement: Document S1. Tables S1–S12 [file mmc1.pdf]

iScience, Volume *28*

Supplemental information

Learning, economies of scale, and knowledge gap effects on power generation technology cost improvements

Yoga W. Pratama, Matthew J. Gidden, Jenna Greene, Andrew Zaiser, Gregory Nemet, and Keywan Riahi

TABLES OF TECHNOLOGY HISTORICAL DATA

Table S1. Nuclear pre-1967 Historical Data, related to Figures 4 and 7 and the STAR Methods[1,2]

Year	Cumulative Capacity (MW)	Cumulative Units (#)	Cumulative Projects (#)	Unit Size (MW/unit)	Project Size (Unit/project)	Capex ($/kW)
1954	60	1.0	1.0	60.0	1.0	6814.0
1956	897	9.0	9.0	115.4	1.0	2502.0
1957	2733	22.0	22.0	141.2	1.0	8512.0
1958	3194	27.0	27.0	92.2	1.0	6294.0
1959	4170	34.0	31.0	139.4	1.7	2889.0
1960	5180	45.0	42.0	91.8	1.0	4221.0
1961	6709	52.0	49.0	218.4	1.0	3742.0
1962	8088	60.0	55.0	172.4	1.3	3594.0
1963	9810	65.0	60.0	344.4	1.0	1959.0
1964	12602	74.0	68.0	310.2	1.1	1822.0
1965	15870	82.0	74.0	408.5	1.3	1696.0
1966	22822	96.0	87.0	496.6	1.1	1608.0

Table S2. Nuclear post-1967 Historical Data, related to Figures 4 and 7 and the STAR Methods[1,2]

Year	Cumulative Capacity (MW)	Cumulative Units (#)	Cumulative Projects (#)	Unit Size (MW/unit)	Project Size (Unit/project)	Capex ($/kW)
1967	39109	61.0	61.0	651.5	1.0	1383.0
1968	65943	98.0	98.0	725.2	1.0	1517.0
1969	75341	111.0	111.0	722.9	1.0	1622.0
1970	100893	148.0	148.0	690.6	1.0	1755.0
1971	113552	166.0	164.0	703.3	1.1	2061.0
1972	134872	194.0	192.0	761.4	1.0	1663.0
1973	159714	224.0	220.0	828.1	1.1	3641.0
1974	195051	262.0	254.0	929.9	1.1	3678.0
1975	231710	300.0	290.0	964.7	1.0	3257.0
1976	273592	343.0	333.0	974.0	1.0	4041.0
1977	295441	366.0	355.0	950.0	1.1	3652.0
1978	317222	389.0	375.0	947.0	1.2	2899.0
1979	340325	416.0	398.0	855.7	1.2	2108.0
1980	359520	436.0	415.0	959.8	1.2	2620.0
1981	375549	453.0	429.0	942.9	1.2	2632.0
1982	394714	471.0	447.0	1064.7	1.0	2676.0
1983	406714	486.0	460.0	800.0	1.2	3037.0
1984	418046	499.0	471.0	871.7	1.1	2241.0
1985	433402	518.0	489.0	808.2	1.1	3274.0
1986	440688	526.0	496.0	910.8	1.1	2997.0
1988	459844	543.0	512.0	1103.1	1.1	3313.0
1989	463862	549.0	517.0	669.7	1.3	2452.0
1990	467129	554.0	522.0	653.4	1.0	3074.0
1991	469375	556.0	524.0	1123.0	1.0	2761.0
1993	476031	563.0	530.0	890.5	1.2	2933.0
1994	477365	565.0	532.0	667.0	1.2	2516.0

Year	Cumulative Capacity (MW)	Cumulative Units (#)	Cumulative Projects (#)	Unit Size (MW/unit)	Project Size (Unit/project)	Capex ($/kW)
1998	484620	575.0	542.0	716.7	1.0	3236.0
2000	494516	588.0	550.0	765.1	1.6	1899.0
2001	495624	589.0	551.0	1108.0	1.0	3071.0
2002	499064	595.0	555.0	573.3	1.6	2324.0
2003	499266	596.0	556.0	202.0	1.0	1897.0
2005	503509	601.0	560.0	969.0	1.0	3823.0
2006	508278	606.0	565.0	953.8	1.0	1979.0

Table S3. Coal Historical Data, related to Figures 4 and 7 and the STAR Methods[3,4]

Year	Cumulative Capacity (MW)	Cumulative Units (#)	Cumulative Projects (#)	Unit Size (MW/unit)	Project Size (Unit/project)	Capex ($/kW)
1927	13163	1266.0	1266.0	10.4	1.0	2068.0
1935	16740	1853.0	1853.0	6.1	1.0	4108.0
1939	18182	1984.0	1984.0	11.0	1.0	2195.0
1940	18763	2061.0	2035.0	7.5	1.5	1914.0
1942	19504	2091.0	2058.0	24.4	1.3	2831.0
1944	20630	2121.0	2087.0	38.7	1.0	2389.0
1945	21238	2166.0	2133.0	13.3	1.0	1767.0
1948	22628	2282.0	2236.0	12.0	1.1	1366.0
1949	23582	2327.0	2273.0	21.1	1.2	1650.0
1950	26534	2471.0	2391.0	20.5	1.2	1647.0
1951	30976	2602.0	2489.0	34.0	1.3	1339.0
1952	31661	2621.0	2501.0	36.7	1.5	1308.0
1953	35315	2685.0	2549.0	56.5	1.3	1300.0
1954	37820	2721.0	2576.0	69.4	1.4	1089.0
1955	47364	2833.0	2641.0	85.8	1.7	1311.0
1956	51001	2898.0	2703.0	56.0	1.0	1195.0
1957	52242	2918.0	2719.0	62.2	1.3	1100.0
1958	52734	2923.0	2723.0	99.4	1.2	1454.0
1959	55524	2949.0	2745.0	104.4	1.2	1073.0
1960	58148	2975.0	2764.0	102.0	1.4	1347.0
1961	59889	3003.0	2786.0	62.5	1.3	1163.0
1962	63846	3051.0	2828.0	81.3	1.2	2387.0
1963	69099	3102.0	2863.0	104.4	1.4	1130.0
1964	73161	3154.0	2906.0	77.9	1.2	1186.0
1965	79017	3207.0	2949.0	109.7	1.2	906.0
1966	88072	3291.0	3013.0	108.1	1.3	1302.0
1967	88628	3296.0	3017.0	112.1	1.3	945.0
1968	99680	3377.0	3077.0	135.9	1.4	692.0
1969	105917	3411.0	3105.0	183.6	1.2	818.0
1970	121976	3484.0	3158.0	221.0	1.4	759.0
1971	134729	3524.0	3191.0	321.4	1.2	895.0
1972	146027	3552.0	3213.0	402.2	1.3	943.0
1973	159609	3592.0	3248.0	333.7	1.2	1421.0
1974	172232	3626.0	3274.0	376.9	1.3	933.0
1975	183127	3682.0	3321.0	196.2	1.2	856.0

Year	Cumulative Capacity (MW)	Cumulative Units (#)	Cumulative Projects (#)	Unit Size (MW/unit)	Project Size (Unit/project)	Capex ($/kW)
1976	192150	3716.0	3349.0	265.1	1.2	1140.0
1977	204288	3746.0	3375.0	402.1	1.2	872.0
1978	212279	3777.0	3402.0	254.9	1.2	1033.0
1979	224068	3816.0	3435.0	301.1	1.2	1250.0
1980	236376	3850.0	3466.0	361.1	1.1	1096.0
1981	245564	3894.0	3505.0	211.1	1.1	1202.0
1982	259153	3960.0	3563.0	204.7	1.1	1311.0
1983	266344	4078.0	3662.0	61.2	1.2	1299.0
1984	274673	4117.0	3696.0	212.0	1.2	1871.0
1985	281150	4175.0	3743.0	111.8	1.2	1541.0
1986	286334	4237.0	3793.0	83.8	1.2	1179.0
1987	288928	4306.0	3849.0	37.6	1.2	1553.0
2000	302216	4474.0	3969.0	78.9	1.4	1642.0
2001	305092	4501.0	3989.0	107.8	1.3	1794.0
2002	305864	4502.0	3991.0	497.0	1.4	2209.0
2003	307804	4514.0	3998.0	169.3	1.5	797.0
2006	311970	4522.0	4004.0	496.4	1.5	2275.0
2007	320238	4536.0	4013.0	590.6	1.5	2244.0

Table S4. Gas OC (Open Cycle) Historical Data, related to Figures 4 and 7 and the STAR Methods[5]

Year	Cumulative Capacity (MW)	Cumulative Units (#)	Cumulative Projects (#)	Unit Size (MW/unit)	Project Size (Unit/project)	Capex ($/kW)
1958	228	1.0	1.0	228.0	1.0	1538.0
1963	5630	30.0	30.0	189.0	1.0	803.0
1969	23434	176.0	176.0	122.0	1.0	726.0
1971	32241	337.0	283.0	54.7	1.5	729.0
1973	44791	555.0	461.0	57.7	1.2	681.0
1974	59446	757.0	589.0	72.4	1.6	608.0
1975	77402	1013.0	682.0	70.3	2.8	602.0
1976	91121	1253.0	785.0	57.0	2.3	587.0
1977	104623	1432.0	874.0	75.6	2.0	588.0
1980	126837	1669.0	968.0	93.6	2.5	578.0
2004	375146	4382.0	2518.0	91.5	1.8	606.4

Table S5. Gas CC (Combined Cycle) Historical Data, related to Figures 4 and 7 and the STAR Methods[6,7]

Year	Cumulative Capacity (MW)	Cumulative Units (#)	Cumulative Projects (#)	Unit Size (MW/unit)	Project Size (Unit/project)	Capex ($/kW)
1981	15736	78.0	58.0	203.0	1.4	1032.0
1982	16271	82.0	60.0	134.6	2.0	1018.0
1983	17318	87.0	63.0	206.7	1.4	907.0
1985	19357	98.0	70.0	183.6	1.7	1137.0
1986	20750	105.0	74.0	214.6	1.8	1184.0
1987	22273	112.0	78.0	204.8	1.7	1014.0
1988	26635	133.0	90.0	213.8	1.7	1247.0
1989	29070	144.0	101.0	212.3	1.0	1032.0
1990	31271	155.0	108.0	208.7	1.4	1267.0
1992	41021	202.0	141.0	204.3	1.5	1196.0
1993	52423	246.0	180.0	260.2	1.1	1051.0
1994	68959	312.0	235.0	252.0	1.2	885.0
1995	82729	367.0	278.0	247.5	1.3	921.0
1996	101846	441.0	330.0	259.2	1.4	851.0
1997	123827	519.0	387.0	282.3	1.4	808.0
2002	317452	940.0	708.0	460.3	1.3	1024.0
2003	395379	1117.0	835.0	439.3	1.4	1049.6
2004	442631	1225.0	918.0	436.1	1.3	1113.6
2005	484604	1346.0	1012.0	348.5	1.3	780.8
2006	525850	1450.0	1095.0	395.5	1.3	960.0
2007	564027	1547.0	1175.0	392.3	1.2	518.4
2008	603749	1640.0	1252.0	428.2	1.2	998.4
2009	647697	1750.0	1341.0	401.5	1.2	947.2
2010	699974	1876.0	1445.0	414.6	1.2	1190.4
2011	745922	1990.0	1537.0	401.4	1.2	1510.4
2012	791672	2101.0	1621.0	412.8	1.3	1433.6

Table S6. Geothermal Historical Data, related to Figures 4 and 7 and the STAR Methods[18–12]

Year	Cumulative Capacity (MW)	Cumulative Units (#)	Cumulative Projects (#)	Unit Size (MW/unit)	Project Size (Unit/project)	Capex ($/kW)
2007	8894	530.0	530.0	16.8	1.0	2119.0
2008	9196	541.0	539.0	27.9	1.3	1995.0
2009	9634	555.0	553.0	29.9	1.0	3991.0
2010	10057	570.0	568.0	28.3	1.0	2630.0
2011	10198	580.0	575.0	14.8	1.5	5338.0
2012	10598	601.0	588.0	19.0	1.6	3842.0
2013	10839	613.0	596.0	20.7	1.3	3627.0
2014	11280	625.0	606.0	35.1	1.3	3552.0
2015	11980	649.0	625.0	29.0	1.3	3730.0
2016	12300	669.0	645.0	16.6	1.0	3890.0
2017	12920	686.0	660.0	35.1	1.2	4237.0
2018	13366	702.0	676.0	28.0	1.0	3978.0

Table S7. Wind Onshore Historical Data, related to Figures 1, 4, and 7 and the STAR Methods[8,9,12–14]

Year	Cumulative Capacity (MW)	Cumulative Units (#)	Cumulative Projects (#)	Unit Size (MW/unit)	Project Size (Unit/project)	Capex ($/kW)
1984	510	14167.0	22.0	0.0	648.1	4950.0
1985	930	23935.0	40.0	0.0	551.8	4850.0
1986	1180	28245.0	50.0	0.1	448.3	4274.0
1987	1360	29896.0	54.0	0.1	385.3	4133.0
1988	1490	30746.0	61.0	0.2	117.6	3174.0
1989	1640	31644.0	65.0	0.2	241.5	2974.0
1990	1840	32629.0	70.0	0.2	183.9	3353.0
1991	2080	33812.0	73.0	0.2	379.3	3299.0
1992	2420	35423.0	87.0	0.2	118.5	3245.0
1993	2900	37474.0	104.0	0.2	123.1	3248.0
1994	3590	39300.0	126.0	0.4	83.3	3007.0
1995	4730	41746.0	173.0	0.5	52.0	2749.0
1996	6025	44362.0	233.0	0.5	43.5	2614.0
1997	7540	47142.0	294.0	0.5	45.5	2473.0
1998	10317	51522.0	419.0	0.6	35.0	2458.0
1999	14355	57366.0	578.0	0.7	36.7	2293.0
2000	19655	63334.0	831.0	0.9	23.6	2241.0
2001	26655	71217.0	1063.0	0.9	34.0	2097.0
2002	33255	76566.0	1369.0	1.2	17.5	2077.0
2003	40755	85012.0	1622.0	0.9	33.4	1976.0
2004	49455	90987.0	1958.0	1.5	17.8	1987.0
2005	60655	98679.0	2292.0	1.5	23.0	1879.0
2006	75355	107798.0	2732.0	1.6	20.7	1915.0
2007	93455	118795.0	3175.0	1.6	24.8	1941.0
2008	117155	133012.0	3708.0	1.7	26.7	2039.0
2009	151155	152530.0	4434.0	1.7	26.9	2073.0
2010	180755	169057.0	5052.0	1.8	26.8	1971.0
2011	218955	188487.0	5784.0	2.0	26.6	1939.0
2012	264355	211817.0	6600.0	1.9	28.6	1995.0
2013	295055	228287.0	7266.0	1.9	24.7	1851.0
2014	343455	253223.0	8211.0	1.9	26.4	1797.0
2015	407855	285326.0	9350.0	2.0	28.2	1659.0
2016	455855	307641.0	10191.0	2.2	26.5	1652.0
2017	498455	325988.0	11038.0	2.3	21.7	1647.0
2018	544455	344918.0	11778.0	2.4	25.6	1566.0
2019	597655	365772.0	12615.0	2.6	24.9	1491.0
2020	703455	404078.0	13900.0	2.8	29.8	1355.0

Table S8. Wind Offshore Historical Data, related to Figures 3, 4, and 7 and the STAR Methods[8,9,12]

Year	Cumulative Capacity (MW)	Cumulative Units (#)	Cumulative Projects (#)	Unit Size (MW/unit)	Project Size (Unit/project)	Capex ($/kW)
2000	40	25.0	6.0	1.6	4.6	2602.0
2001	80	45.0	8.0	2.0	12.5	2424.0

Year	Cumulative Capacity (MW)	Cumulative Units (#)	Cumulative Projects (#)	Unit Size (MW/unit)	Project Size (Unit/project)	Capex ($/kW)
2002	240	125.0	9.0	2.0	80.0	3494.0
2003	456	219.0	11.0	2.3	36.0	2789.0
2004	617	289.0	18.0	2.3	9.9	3170.0
2005	707	319.0	19.0	3.0	30.0	2462.0
2006	800	350.0	20.0	3.0	30.0	2775.0
2007	1108	457.0	24.0	2.9	29.4	4524.0
2008	1228	517.0	25.0	2.0	60.0	5535.0
2009	1578	611.0	28.0	3.7	29.6	4473.0
2010	3059	1122.0	39.0	2.9	46.8	4724.0
2011	3308	1197.0	42.0	3.3	25.2	5591.0
2012	3796	1311.0	44.0	4.3	56.0	4212.0
2013	6762	2052.0	56.0	4.0	62.2	5346.0
2014	7831	2367.0	61.0	3.4	59.2	5887.0
2015	11780	3307.0	78.0	4.2	55.1	4718.0
2016	13104	3572.0	87.0	5.0	29.8	3596.0
2017	16502	4238.0	101.0	5.1	49.9	4565.0
2018	21094	4979.0	120.0	6.2	37.9	3942.0
2019	26942	5826.0	146.0	6.9	32.8	3614.0

Table S9. Solar PV (Utility) Historical Data, related to Figures 4, and 7 and the STAR Methods[8,9,12,15,16]

Year	Cumulative Capacity (MW)	Cumulative Units (#)	Cumulative Projects (#)	Unit Size (MW/unit)	Project Size (Unit/project)	Capex ($/kW)
2010	7200	24000001.0	216.0	0.0003	111400.0	4778.0
2011	17500	58333334.0	488.0	0.0003	126131.3	3999.0
2012	29100	97000001.0	790.0	0.0003	128038.2	3032.0
2013	50200	167333334.0	1286.0	0.0003	141972.8	2657.0
2014	75400	251333334.0	1865.0	0.0003	145070.4	2402.0
2015	108100	360333334.0	2622.0	0.0003	143996.0	1830.0
2016	165200	550666667.0	3854.0	0.0003	154398.5	1663.0
2017	226300	754333334.0	5162.0	0.0003	155724.9	1437.0
2018	281500	938333334.0	5888.0	0.0003	253541.8	1228.0
2019	347000	1156666667.0	6745.0	0.0003	254704.6	1011.0
2020	421200	1404000000.0	7556.0	0.0003	305126.1	890.0
2021	498600	1662000000.0	8362.0	0.0003	320095.6	833.0

Table S10. Solar CSP Historical Data, related to Figures 4, and 7 and the STAR Methods[8,9,12,17,18]

Year	Cumulative Capacity (MW)	Cumulative Units (#)	Cumulative Projects (#)	Unit Size (MW/unit)	Project Size (Unit/project)	Capex ($/kW)
1985	30	1.0	1.0	30.0	1.0	19564.0
1986	90	3.0	2.0	30.0	2.0	13912.0
1987	120	4.0	3.0	30.0	1.0	13260.0
1988	180	6.0	4.0	30.0	2.0	14565.0

1989	260	7.0	5.0	80.0	1.0	16956.0
1990	340	8.0	6.0	80.0	1.0	14565.0
2007	412	9.0	7.0	72.0	1.0	14999.0
2008	462	17.0	15.0	7.0	1.0	11739.0
2009	782	24.0	20.0	46.0	1.4	10434.0
2010	1282	31.0	26.0	71.0	1.2	9130.0
2011	1682	39.0	33.0	50.0	1.1	10757.0
2012	2582	51.0	44.0	75.0	1.1	8313.0
2013	3882	61.0	52.0	130.0	1.3	6522.0
2014	4482	66.0	56.0	120.0	1.3	5598.0
2015	4682	68.0	58.0	100.0	1.0	7479.0
2016	4982	73.0	63.0	60.0	1.0	7861.0
2017	5082	74.0	64.0	100.0	1.0	7441.0
2018	5782	81.0	70.0	100.0	1.1	5337.0
2019	6382	90.0	79.0	67.0	1.0	6686.0
2020	6582	92.0	81.0	100.0	1.0	4599.0
2021	6682	93.0	82.0	100.0	1.0	8809.0

Table S11. Wind Offshore Depth Historical Data, related to Figure 3[19]

No.	Year	Water Depth (m)
1	2001	6
2	2002	11
3	2003	13
4	2003	9
5	2004	12
6	2004	10
7	2005	4
8	2006	16
9	2007	18
10	2007	13
11	2008	24
12	2009	20
13	2009	16
14	2009	11
15	2009	8
16	2010	17
17	2010	30
18	2010	20
19	2010	23
20	2010	12
21	2011	23
22	2011	19
23	2012	30
24	2012	21
25	2013	41
26	2013	37
27	2013	17
28	2013	12
29	2013	23

No.	Year	Water Depth (m)
30	2013	20
31	2014	23
32	2014	18
33	2014	27
34	2014	21
35	2015	25
36	2015	41
37	2015	32
38	2015	29
39	2015	21
40	2015	16
41	2016	28
42	2016	34
43	2017	41
44	2017	33
45	2017	24
46	2017	14
47	2017	11
48	2017	29
49	2018	54
50	2018	39
51	2018	34
52	2018	23
53	2019	40
54	2019	50
55	2019	37
56	2019	26
57	2019	19
58	2019	33
59	2019	28
60	2020	30
61	2020	40
62	2020	30
63	2020	26
64	2020	37
65	2020	33
66	2020	38
67	2020	41
68	2018	29
69	2018	30
70	2018	40
71	2017	34
72	2015	22
73	2013	22
74	2013	20
75	2015	34
76	2015	10
77	2015	10
78	2010	10
79	2016	13

No.	Year	Water Depth (m)
80	2016	8
81	2016	5
82	2017	12
83	2018	14
84	2018	12
85	2019	15
86	2019	13
87	2019	6
88	2019	5
89	2020	29
90	2020	28
91	2020	24
92	2020	20
93	2020	13
94	2020	11
95	2020	10
96	2020	9

Table S12. Wind Offshore Distance to Shore Historical Data, related to Figure 3[19]

No.	Year	Distance to Shore (km)
1	2001	4.3
2	2002	17.2
3	2003	11.2
4	2003	4.4
5	2004	8.8
6	2004	11.6
7	2005	9.3
8	2006	12.7
9	2007	13.2
10	2007	8.8
11	2008	25.9
12	2009	27.4
13	2009	9.2
14	2009	11.3
15	2009	6
16	2010	55.6
17	2010	44.5
18	2010	32
19	2010	8.6
20	2011	16.3
21	2012	21.9
22	2012	11.9
23	2013	21.1
24	2013	111.8
25	2013	31.9
26	2013	27.4

No.	Year	Distance to Shore (km)
27	2013	6.5
28	2013	23
29	2013	9
30	2014	54.5
31	2014	41.7
32	2014	35.8
33	2014	20
34	2014	2.8
35	2015	109.1
36	2015	74.2
37	2015	65.6
38	2015	55.6
39	2015	45.1
40	2015	34.8
41	2015	24.5
42	2015	17.6
43	2015	9.7
44	2015	51.3
45	2016	44.2
46	2016	28
47	2017	114
48	2017	97.3
49	2017	69.8
50	2017	37.5
51	2017	16.5
52	2017	10.8
53	2018	31.1
54	2018	16.6
55	2018	2.7
56	2018	56.6
57	2018	39.3
58	2018	35.1
59	2018	28
60	2019	114.3
61	2019	104.5
62	2019	60.7
63	2019	37.1
64	2019	18.7
65	2019	24.2
66	2020	51.8
67	2020	111.4
68	2020	65.4
69	2020	38.4
70	2020	31
71	2020	55.2
72	2020	47.5
73	2019	117.6
74	2020	40.2
75	2019	31.3

No.	Year	Distance to Shore (km)
76	2018	6.3
77	2017	43.2
78	2010	17.6
79	2010	15
80	2011	19.8
81	2015	13.7
82	2015	11.1
83	2016	23.5
84	2016	10.5
85	2016	6.4
86	2017	9.8
87	2018	23.1
88	2018	43.4
89	2019	48.7
90	2019	26.1
91	2019	23.5
92	2019	20.2
93	2020	12.7
94	2020	22
95	2020	24.4
96	2020	25.2
97	2020	29.3
98	2020	43.9
99	2020	42.1

REFERENCES

1. Lovering, J. R., Yip, A. & Nordhaus, T. Historical construction costs of global nuclear power reactors. *Energy Policy* **91**, 371–382 (2016).
2. IAEA. *Nuclear Power Reactors in the World*. (International Atomic Energy Agency - IAEA, Vienna, 2022).
3. McNerney, J., Doyne Farmer, J. & Trancik, J. E. Historical costs of coal-fired electricity and implications for the future. *Energy Policy* **39**, 3042–3054 (2011).
4. Yeh, S. & Rubin, E. S. A centurial history of technological change and learning curves for pulverized coal-fired utility boilers. *Energy* **32**, 1996–2005 (2007).
5. Rogner, H. Hydrogen technologies and the technology learning curve. *Int. J. Hydrog. Energy* **23**, 833–840 (1998).
6. Colpier, U. C. & Cornland, D. The economics of the combined cycle gas turbine—an experience curve analysis. *Energy Policy* **30**, 309–316 (2002).

7. International Energy Agency. *Experience Curves for Energy Technology Policy*. (OECD, 2000). doi:10.1787/9789264182165-en.
8. IRENA. Renewable power generation costs in 2021. (2022).
9. IRENA. *Renewable Power Generation Costs in 2022*. (Abu Dhabi, 2023).
10. Ediger, V. Ş. & Akar, S. Historical Pattern Analysis of Global Geothermal Power Capacity Development: Preprint. *Renew. Energy* (2023).
11. Barbier, E. Geothermal energy technology and current status: an overview. *Renew. Sustain. Energy Rev.* **6**, 3–65 (2002).
12. International Energy Agency. Renewable Energy Progress Tracker. (2023).
13. *Renewable Energy Sources and Climate Change Mitigation: Summary for Policymakers and Technical Summary*. (International Panel of Climate Change, Geneva, 2011).
14. Wiser, R. *et al. Land-Based Wind Market Report: 2023 Edition*. (2023).
15. Barbose, G., Darghouth, N. & Wiser, R. *Tracking the Sun V: An Historical Summary of the Installed Price of Photovoltaics in the United States from 1998 to 2011*. LBNL--5919E, 1172692 http://www.osti.gov/servlets/purl/1172692/ (2012) doi:10.2172/1172692.
16. Barbose, Galen & Darghouth, Naim. *Tracking the Sun: Pricing and Design Trends for Distributed Photovoltaic Systems in the United States - 2019 Edition*. https://escholarship.org/uc/item/5422n7wm (2019).
17. Lilliestam, J., Ollier, L., Labordena, M., Pfenninger, S. & Thonig, R. The near- to mid-term outlook for concentrating solar power: mostly cloudy, chance of sun. *Energy Sources Part B Econ. Plan. Policy* **16**, 23–41 (2021).
18. Thonig, R., Gilmanova, A. & Lilliestam, J. CSP.guru 2023-07-01. Zenodo https://doi.org/10.5281/ZENODO.1318151 (2023).
19. Musial, W. *et al. Offshore Wind Market Report: 2023 Edition*. https://tinyurl.com/m475zt3k (2023).
